# Impact of Unilateral Sciatica Due to Lumbar Disc Hernia on Gait

**DOI:** 10.3390/healthcare14101268

**Published:** 2026-05-07

**Authors:** Patricia Balestra-Romero, María Reina-Bueno, María del Carmen Vázquez-Bautista, Pedro V. Munuera-Martínez, Inmaculada C. Palomo-Toucedo

**Affiliations:** Department of Podiatry, Faculty of Nursing, Physiotherapy and Podiatry, Universidad de Sevilla, 41009 Seville, Spain; patbalrom@alum.us.es (P.B.-R.); carmenvaz@us.es (M.d.C.V.-B.); pmunuera@us.es (P.V.M.-M.); ipalomo@us.es (I.C.P.-T.)

**Keywords:** sciatica, lumbar disc herniation, chronic radicular pain, gait analysis, plantar pressure, radicular low back pain

## Abstract

**Background/Objectives**: Sciatica secondary to lumbar disc herniation is a common cause of chronic radicular pain and functional disability. Since the sciatic nerve is involved in the motor and sensory innervation of the foot, it is important to evaluate the potential distal biomechanical alterations it produces. Evidence regarding the effect of radicular pain on kinetic parameters remains limited and heterogeneous. The aim of this study was to describe gait characteristics in people with chronic unilateral radicular pain due to non-traumatic lumbar or lumbosacral disc herniation and to compare kinetic differences between the affected and unaffected limbs. **Methods**: A cross-sectional analytical observational study was conducted in 41 patients who met the inclusion criteria. Dynamic baropodometric assessment was performed using the Footscan^®^ system. The analysis focused on kinetic parameters, including surface area, pressure, and maximum force, as well as spatiotemporal variables comprising stance time, step time, step length, and plantar push-off mechanics. Demographic data, Foot Posture Index (FPI) scores, and muscle strength were also recorded. **Results**: According to patient reports, the left foot was the most severely affected. Significant differences in muscle strength were found between the affected and unaffected limbs. However, no significant differences were observed in any of the kinetic or spatiotemporal parameters evaluated. **Conclusions**: Patients with unilateral sciatica due to lumbar disc herniation showed reduced muscle strength in the affected limb with no significant differences in kinetic or spatiotemporal gait parameters, suggesting compensatory mechanisms.

## 1. Introduction

Radicular lumbar or lumbosacral pain, also known as sciatica, is caused by overstimulation of the sciatic nerve, which is formed by nerve roots arising from L4 to S3 [1]. It is characterised by pain in one or more lumbar or sacral dermatomes and may be accompanied by radicular irritative symptoms and functional deficits [2]. It may result from any condition that structurally affects or compresses the sciatic nerve, with lumbar disc herniation being the most common cause [1,3].

Estimates of the prevalence of sciatica vary widely due to a lack of consensus on its definition and methodological differences between studies [4]. In the general population, annual prevalence ranges from 9.9% to 25%, while lifetime prevalence ranges from 1.2% to 43% [2]. 

Sciatica is a highly debilitating condition with a significant impact on quality of life and substantial economic and social burdens [5]. Compared with non-specific low back pain, it is associated with greater pain and disability, as well as increased use and cost of healthcare services, including lost workdays [6,7]. Identifying new conservative treatment options may help reduce the social and personal impact of an individual’s health condition. Gait analysis provides useful insights to guide podiatric interventions.

The sciatic nerve provides direct motor innervation to the hamstring muscles and indirect motor innervation to the calf muscles, the anterior compartment of the leg, and certain intrinsic foot muscles, while its terminal branches provide sensory innervation to the sole of the foot and the posterior and lateral regions of the leg [1]. In particular, all distal motor and sensory function [8] are mediated primarily by the L5 and S1 roots.

Although unilateral sciatica was not specified as an inclusion criterion in any study, joint and muscle differences have been reported in people with sciatica compared with healthy individuals [9], suggesting altered distal motor control during gait. Patients with sciatica exhibited greater instability during both the stance and swing phases, which was associated with pain intensity [10] and functional limitations [11]. In particular, patients with sciatica secondary to lumbar disc herniation showed slower gait speed, shorter step length, and longer foot contact time during the stance phase [12]. When attempting to walk faster, they tended to modify cadence before step length, a pattern that has also been observed in patients with non-specific low back pain. [13]. 

Lumbar motion was restricted between the upper lumbar segments (L1–L3) and the lower lumbar segments (L3–L5), with compensatory movements occurring at the knee, hip, and pelvis, including increased pelvic rotation, as well as alterations in intersegmental coordination, particularly between the lumbar segments and between the thorax and pelvis during functional activities [14]. Distal alterations were also observed at the foot. These included abnormally reduced subtalar joint motion and decreased hallux extension. In addition, plantar pressure distribution was abnormal, with imbalanced loading across specific plantar regions and increased loading on the lateral metatarsals and lesser toes during push-off [12]. Collectively, these adaptations resulted in an asymmetric and unstable gait pattern [10,15], affecting both proximal and distal components. Some authors considered the changes observed to be a compensatory strategy to minimise pain and neural irritation [12,14].

However, this is not the case in patients with sciatica due to lumbar disc herniation. The relationships between lumbopelvic alterations and foot kinetics during walking have been thoroughly explored in patients with non-specific low back pain [16,17]; in contrast, the influence of the sciatic nerve on these same parameters has not been sufficiently investigated in patients with sciatica due to lumbar disc herniation. This comparison would enable a more accurate quantification of compensatory strategies, providing clearer insight of how localised pathology disrupts global motor control.

The main objective of this study was to describe some gait characteristics of people with chronic radicular pain secondary to unilateral, non-traumatic lumbar or lumbosacral disc herniation and to compare kinetic differences between the affected and unaffected limbs. Gait asymmetry analysis may guide conservative interventions, such as podiatric management and gait retraining, to reduce pain and identify the risk of secondary injury in the unaffected limb due to compensatory overload. Comparing affected and unaffected limbs enables precise quantification of compensatory strategies, providing insight into how localised pathology alters global motor control.

## 2. Materials and Methods

A cross-sectional analytical observational study was conducted. The study was submitted for ethical review through the Biomedical Research Ethics Portal of Andalusia (PEIBA) and received approval from the Clinical Research Ethics Committee of University Hospital Nuestra Señora de Valme (Internal Code: 2449-N-21).

The inclusion criteria were age between 18 and 70 years and a medical diagnosis, confirmed by a magnetic resonance imaging (MRI) report, of chronic radicular low back pain caused by unilateral non-traumatic lumbar or lumbosacral disc herniation. Individuals with a history of previous lumbar surgery or osteoarticular foot surgery, neuromuscular or joint disorders of the foot, other spinal pathologies, neuropathic pain related to diabetes mellitus or other conditions, pregnancy, cognitive impairment, or concomitant chronic rheumatic disease were excluded.

The sample was obtained through convenience sampling based on predefined clinical criteria. Patients were recruited by two specialists in Anaesthesiology and Resuscitation from the Chronic Pain Unit of the Southern Seville Health Management Area, part of the Andalusian Health System. These specialists informed patients diagnosed with non-traumatic lumbar or lumbosacral disc herniation about the nature of the study. Only the first names and telephone numbers of those who agreed to participate were provided to the research team. Two members of the research team then contacted the patients by telephone to arrange their participation in the study. Of the 98 patients contacted, 51 were excluded for a range of clinical, functional, and logistical reasons, and 47 met the eligibility criteria and agreed to participate. Of these, 6 were excluded during the final assessment because they did not have unilateral involvement, resulting in a final sample of 41 participants. All patients were verbally informed about the voluntary nature of the study, its characteristics, and provided written informed consent. After signing the informed consent form, they were evaluated at the Podiatry Clinic of the University of Seville between March 2022 and May 2024. Demographic data were recorded, including age, sex, weight, and height, which were used to calculate body mass index (BMI), as well as the laterality of the affected limb.

A muscle examination of the foot was then performed. Muscle strength of the extrinsic muscles (located on the anterior, posterior, medial, and lateral aspects of the leg) was evaluated using manual muscle testing according to the Medical Research Council (MRC) Scale of Muscle Strength. This method assesses strength on an ordinal scale from 0 to 5 (0 = no contraction; 5 = normal strength) [18]. A dynamometer was also used [19], which provides a quantitative measure of muscle strength on a continuous scale from 0 to 100. Three measurements were taken, and the mean of the three measurements was calculated from the recorded maximum and minimum values. Only registers of tibialis anterior and gluteus medius were performed by dynamometry.

The weight-bearing foot assessment was performed with the patient standing on a dedicated platform. The Foot Posture Index [20], a validated clinical tool based on six inspection and palpation criteria of the weight-bearing foot, was then applied. This tool allows the foot posture to be classified through the evaluation of six anatomical and functional criteria. Each item is scored on an ordinal scale from −2 (supination) to +2 (pronation), yielding a total score ranging from −12 to +12. Based on this score, feet are classified as supinated (−12 to −1), neutral (0 to +5), or pronated (+6 to +12). This approach enables standardised comparison between participants and facilitates the monitoring of foot posture. Static and dynamic plantar pressure analyses were performed using the Footscan^®^ platform (RSscan International, Olen, Belgium). The reliability and repeatability of this system have previously been demonstrated [21]. The platform measured 2093 × 469 × 18 mm and incorporated 16,384 resistive sensors arranged in a 256 × 64 matrix. It has a resolution of 4 sensors/cm^2^, an active sensor area of 1950 × 325 mm, a data acquisition frequency of 125 Hz, and a pressure range of 1–200 N/cm^2^.

The platform was installed flush with the ground within a wooden walkway measuring 2 m in width and 10 m in length. These dimensions allowed the simulation of a normal walking surface and minimised targeting or step adaptation during dynamic analysis. Following the manufacturer’s instructions, the system was calibrated before data collection by entering the patient’s body weight and foot size into the software. Participants were then first asked to stand in a comfortable position and then walk across the platform. The software calculated the recalibration factor for future measurements in the same individual.

The platform was connected to a computer that transmitted and processed the data using Scientific Foot-Scan^®^ software (RSscan International, Olen, Bélgica, V9). This system automatically divided the foot into three main regions (heel, midfoot, and forefoot) or into ten anatomical zones (medial heel, lateral heel, midfoot, metatarsals 1–5, hallux, and lesser toes), depending on the variables analysed. For the statistical analysis, surface area (%), pressure (N/cm^2^), and stance time (ms) were selected for each region. The maximum force (N) and maximum pressure (N/cm^2^) were also identified for the different zones. In addition, step time (s) and step length (mm) were recorded [22]. Maximum values were obtained by identifying the peak of each force–time curve. Impulse (the force–time integral), which represents the cumulative effect of plantar force during the stance phase of gait, was calculated to characterise plantar loading in the anatomical regions and the dynamic function of the foot. This allowed the percentage of local impulse to be calculated for three regions, namely the rearfoot, midfoot, and forefoot [23], while normalising the data for body weight variability and demonstrating high reliability (intraclass correlation coefficient >0.89) [24].

Gait data were obtained following the acclimatisation protocol described by Xu et al. [21], which involved a 10 min familiarisation walk on the measurement surface. This procedure allowed individual standardisation of stride length and established a starting point that ensured three successive steps before contact with the platform, thereby enabling the recording of data representative of average gait and minimising the effects of acceleration or deceleration. Data were collected during five complete walks across the platform. Subsequently, two experienced researchers selected the three most consistent footprints from each foot for final analysis (complete impressions, showing an initial heel-to-toe contact pattern and no forced adjustments in the patient’s gait). Finally, the values for each variable were calculated as the mean of the three selected recordings. All examinations were performed by the same researchers.

Statistical analysis of the data was performed using IBM SPSS Statistics v26 (IBM, Armonk, NY, USA), available under licence from the University of Seville. The recorded variables included categorical and nominal variables, for which absolute frequencies (N) and relative frequencies (%) were calculated. The values of affected and unaffected members were compared using statistical tests to determine if there were significant differences. For quantitative variables, the mean and standard deviation were calculated. The normality of the quantitative variables was assessed using the Shapiro–Wilk test. The Forefoot Surface Area %, impulse percentage parameters, and dynamometer-based strength measurements followed a normal distribution (*p* > 0.05). Conversely, the FPI, manual muscle testing, and remaining pressure and gait variables did not follow a normal distribution (*p* < 0.05). Based on these results, to compare both limbs, the paired t-test was used when the assumption of normality was met, whereas the Wilcoxon signed-rank test was used when the data were not normally distributed. For categorical variables, comparisons were made using Pearson’s chi-squared test. *p*-values of less than 0.05 were considered statistically significant. Effect size (Cohen’s d) was calculated for variables showing statistically significant differences (*p* < 0.05) and interpreted as follows: <0.10 very small; 0.10–0.29 small; 0.30–0.49 moderate; and ≥0.50 large.

Data monitoring was conducted in collaboration with an external statistician, who also reviewed all aspects of data collection and analysis throughout the study.

## 3. Results

The sample had a mean age of 46.05 ± 9.36 years, a balanced sex distribution, and a mean body mass index of 26.99 ± 4.24. The left limb was affected in 65.9% of participants (*n* = 27), whereas the right limb was affected less frequently (34.1%; *n* = 14), with a statistically significant difference in laterality (*p* = 0.004). In addition, 75.6% of participants did not have a certified degree of disability.

Table 1 shows the FPI values and muscle strength findings. Statistically significant differences were observed in supinator muscle strength and in the mean and maximum dynamometry values for the gluteus medius and tibialis anterior, with lower values observed in the affected limb compared to the unaffected limb. The effect size was large in the gluteus medius and moderate in the plantar flexors, supinators, tibialis anterior, tibialis anterior maximus, and gluteus medius maximus.

No statistically significant differences were found in the spatiotemporal gait parameters between the affected and unaffected limbs, as shown in Table 2.

The step time for the affected limb was 201.38 ± 611.94 s (mean ± standard deviation [SD]), while the unaffected limb was 46.62 ± 238.50 s. The step length for the affected limb was 566.23 ± 314.27 s, while the unaffected limb was 463.99 ± 93.24 s.

Regarding pressure distribution, rearfoot pressure was 23.17 ± 8.60 N/cm^2^ in the affected limb and 23.80 ± 8.16 N/cm^2^ in the unaffected limb. The midfoot pressure was 8.53 ± 4.78 N/cm^2^ on the affected limb and 8.93 ± 4.6 N/cm^2^ on the unaffected limb. Additionally, forefoot pressure was 18.06 ± 7.00 N/cm^2^ and 18.60 ± 6.77 N/cm^2^ (SD) for the affected and unaffected limbs, respectively.

Regarding the impulse distribution per zone, the affected limb showed a rearfoot impulse percentage of 10.46 ± 3.87%, a midfoot impulse percentage of 24.45 ± 12.26%, and a forefoot impulse percentage of 65.09 ± 12.19%. For the unaffected limb, these values were 10.03 ± 3.79% for the rearfoot, 23.79 ± 12.99% for the midfoot, and 66.18 ± 13.91% for the forefoot impulse percentage.

## 4. Discussion

The main objective of this study was to analyse differences in spatiotemporal and biomechanical gait parameters between the affected and unaffected limbs in people with chronic radicular pain secondary to unilateral, non-traumatic lumbar or lumbosacral disc herniation.

The results showed that the affected limb exhibited significantly lower strength in the gluteus medius and supinator muscles, especially the tibialis anterior, compared with the unaffected limb. Despite this, no differences were observed in foot posture or gait biomechanical patterns between the two limbs, suggesting that the observed weakness might be more closely related to perceived pain than to distal neuromuscular injury. In addition, compensatory mechanisms may be activated to reduce asymmetry during walking to preserve gait function and efficiency.

The significant weakness of the gluteus medius supports the hypothesis proposed by Hatakeyama et al. [25], who studied a cohort of 84 patients diagnosed with lumbar disc herniation or lumbar spinal stenosis with unilateral symptoms. In their study, they observed a significant reduction in hip abductor muscle strength as measured by digital dynamometry and concluded that this proximal muscle was a highly sensitive indicator for quantifying L5 nerve root involvement.

On the other hand, Dove et al. [26] observed that, although 85% of patients with unilateral sciatica reported subjective weakness, only 34% showed an objective motor deficit when assessed using the Medical Research Council Scale. According to the authors, this discrepancy between patient perception and clinical findings appeared to be mediated primarily by the severity of radicular pain. These results suggest that the expected functional deficit may not correspond to irreversible structural motor damage, but rather to a strategy aimed at avoiding neuropathic pain and reflecting reduced confidence in physical stability. This interpretation is also supported by the findings of Elbaz et al. [13], who studied a cohort of patients with non-specific chronic low back pain using an electronic platform to measure various spatiotemporal parameters. In their study, they found that those patients adopted a generally more restrictive motor pattern, characterised by slower speed and shorter step length as a protective mechanism to minimise the forces acting on the body.

Wang et al. [27], studied patients with herniated discs and healthy controls to assess the percentage of fat accumulated within muscle tissue using a magnetic resonance imaging technique known as q-Dixon MRI. Their study suggested that measuring fat infiltration via this technique offered a far more sensitive indicator of muscle degeneration than muscle size. The authors found significantly higher levels of fat infiltration in the gluteus medius and paravertebral muscles of patients with herniated discs, which could explain the persistent reduction in contractile capacity after years of disease. This reduction in contractile capacity could also explain the discrepancy between our results and those of similar studies, since our sample consisted of patients referred from a chronic pain unit with a long history of the condition.

The absence of statistically significant differences in baropodometric and spatiotemporal parameters between the affected and unaffected lower limbs was the central finding of our study. This symmetrical pattern of load distribution, plantar pressure, and stance time challenged the clinical expectation of a lateralised biomechanical deficit, even in the context of high pain intensity and chronicity. Local treatment strategies such as functional rehabilitation or customised foot orthoses may therefore provide limited clinical benefits.

Strutton et al. [28], demonstrated, using transcranial magnetic stimulation, that patients with chronic unilateral sciatica exhibited a bilateral reduction in corticospinal excitability, with no differences between the affected and contralateral limbs. This finding suggested that the adaptation was not purely peripheral but also involved central mechanisms. In their review, Meier et al. [29] stated that the nervous system prioritised stability over mechanical efficiency in the presence of persistent pain. Similar findings were reported by Korkusuz et al. [30], who compared healthy individuals, patients with mechanical low back pain, and patients with neuropathic low back pain associated with disc degeneration. They found no significant differences in speed, cadence, or the dynamic distribution of plantar pressure between the affected and unaffected limbs in patients with either type of low back pain.

In their review, Natarajan et al. [22] conducted a qualitative synthesis of spatial and temporal gait metrics to identify specific gait profiles in various degenerative lumbar pathologies. They reported that gait in patients with lumbar disc herniation was characterised by reduced speed and cadence, together with increased double-support time and greater step variability. The authors concluded [22,30] that the motor reorganisation associated with chronic pain did not act solely on the affected segment, but rather globally modulated motor control in both lower limbs, thereby minimising neural irritation and enhancing gait stability.

Frost et al. [31] studied a cohort of patients with chronic low back pain due to unilateral radiculopathy, together with a group of healthy participants. A force platform was used to analyse various balance-related parameters, and electromyography was used to assess the activation timing and response sequence of several muscle groups. Their results suggested that patients with sciatica tended to activate multiple muscle groups simultaneously. This resulted in a more rigid and less selective activation pattern. In contrast, healthy individuals showed a sequential pattern of muscle activation from proximal to distal. However, they found no differences in the remaining parameters, which they attributed to the moderate degree of disability in the sample.

Roelker et al. [32] studied the influence of the supporting limb on the position of the swinging limb in a small sample of healthy volunteers. Their objective was to analyse the individual muscular contributions to foot placement during gait. Based on data collected using an instrumented rolling platform, electromyography, and advanced biomechanical simulations, they concluded that gait control did not depend on a single muscle. Instead, it relied on multisegmental coordination capable of making adjustments in proximal segments or in the contralateral limb to compensate for local deficits.

Both studies reinforced the idea that simultaneous muscle activation and intersegmental coordination strategies may be involved. Together with the motor reorganisation associated with chronic pain [22,30], this may represent an adaptive phenomenon leading to a more balanced gait. Such adaptation may help to minimise impact forces and distribute loads more evenly between both limbs. This could explain the absence of asymmetries in the other parameters studied.

In contrast, some studies have described plantar asymmetries in specific subgroups of patients with lumbar radiculopathy. Lee et al. [10] explored how pain intensity influenced gait symmetry in patients with lumbar disc herniation using a photoelectric cell system to quantify spatiotemporal parameters. They calculated the gait symmetry index to assess variability and balance between both limbs. Based on this analysis, they concluded that pain intensity was associated with gait symmetry in both the stance and swing phases.

Wang et al. [15] studied patients with lumbar disc herniation and unilateral pain, comparing them with healthy individuals both before and after surgery. They calculated an asymmetry index to assess various spatiotemporal and kinetic parameters collected using a force platform, a motion capture system, and biomechanical simulation. This analysis compared the affected and unaffected limbs in both groups. They concluded that patients with lumbar disc herniation showed significantly greater asymmetry in gait cycle time and step length than healthy individuals.

Kanna et al. [8] analysed various static and dynamic baropodometric parameters in patients with unilateral L5–S1 radiculopathy of different aetiologies, compared with a control group. They observed a significant reduction in load transmission in the foot on the affected side compared with healthy individuals, assessed before surgical intervention.

The discrepancy between these findings and ours could be explained by the severity and duration of the clinical presentation. Several of these studies included patients with a shorter duration of symptoms. By contrast, our sample showed marked chronicity, with a disease duration of at least 3 years, which may have favoured the development of more stable bilateral compensatory strategies. Similarly, the sample studied by Kanna et al. [8] included other radiculopathies, such as lumbar stenosis, in which gait has been described as asymmetric and variable [22]. This may also have contributed to the observed differences.

Our study had several limitations. The use of self-selected gait speed, although consistent with the protocol described by Xu et al. [21], may have contributed to the attenuation of asymmetries that become apparent under greater functional demands. Furthermore, the sample was not randomised, which limited the generalisability of the findings and reduced the ability to detect smaller differences. The higher prevalence of left lower limb involvement, together with the lack of information on limb dominance, prevented the analysis of possible relationships between functional dominance and the affected side. Although manual dynamometry has acceptable clinical reliability, the assessment of muscle strength is less sensitive than instrumented methods. Another limitation of our study is that we did not compare muscle weakness in affected and unaffected limbs on an individual basis due to the potential heterogeneity of compensatory mechanisms. Finally, it remains unknown whether these findings would also apply to patients with the same diagnosis but with acute symptoms or no symptoms at all.

Taken together, our findings suggest that not all patients with sciatica develop a unilateral alteration in podiatric function. In the presence of severe and chronic pain, bilateral stiffness and motor control strategies may predominate, while the most relevant alterations may occur in proximal segments rather than at the plantar level. In this context, our results indicate that podiatric and gait abnormalities were not significantly associated with pain chronicity, in contrast to what has been described in non-specific low back pain. From this perspective, the routine indication for podiatric examination or intervention in these patients may not be justified. If podiatric abnormalities are identified, and although symptoms may be more severe in the affected limb, our findings would support a symmetrical therapeutic approach rather than strategies focused exclusively on the symptomatic side.

## 5. Conclusions

Patients with unilateral sciatica secondary to lumbar disc herniation showed significantly reduced muscle strength in the affected limb compared with the unaffected limb, particularly in the gluteus medius and tibialis anterior. However, no significant differences were observed in the kinetic and spatiotemporal gait parameters evaluated. These findings may suggest that sciatica is not necessarily associated with an altered unilateral gait pattern and that the predominant functional adaptations remain unclear. Further investigation is therefore warranted for both bilateral adaptations and a potential central origin, as well as gait compensatory mechanisms, given their potential clinical implications for comprehensive functional rehabilitation and podiatric treatment.

## Figures and Tables

**Table 1:** FPI and muscle examination of the affected and unaffected limbs.

Variable	Affected Limb (*n* = 41)		Unaffected Limb (*n* = 41)		Size Effect	*p*
	Mean	SD	Mean	SD		
**Foot Posture Index**	2.56	4.00	2.32	3.18	-	0.642
**Tibialis anterior ^#^** **(MRC)**	4.73	0.55	4.83	0.44	-	0.102
**Gluteus medius ^#^** **(MRC)**	4.28	1.03	4.53	0.81	-	0.160
**Dorsalis flexors ^#^** **(MRC)**	4.76	0.49	4.88	0.33	-	0.059
**Plantar flexors ^#^** **(MRC)**	4.85	0.36	4.95	0.22	-	0.102
**Pronators ^#^** **(MRC)**	4.51	0.68	4.68	0.52	-	0.052
**Supinators ^#^** **(MRC)**	4.54	0.64	4.73	0.55	-	0.005
**Anterior tibial strength** **(dynamometer)**	37.69	12.10	43.54	13.15	0.463	0.003
**Maximum anterior tibial strength (dynamometer)**	58.40	15.74	64.93	18.97	0.375	0.001
**Gluteus medius strength** **(dynamometer)**	27.77	15.44	36.42	17.08	0.531	<0.001
**Maximum gluteus medius strength (dynamometer)**	46.83	22.28	58.37	25.83	0.478	<0.001

^#^ Scores ranged from 0 to 5 (muscle strength quantified with manual muscle testing according to the Medical Research Council Muscle Strength Scale). Comparisons for dynamometry variables were performed using the paired *t*-test, while the Wilcoxon signed-rank test was used for the Foot Posture Index and MRC scales. MRC values = (0–5). FPI values (−12–+12).

**Table 2:** Comparison of spatiotemporal gait parameters and plantar pressure distribution between limbs.

Variable	Affected Limb (*n* = 41)			Unaffected Limb (*n* = 41)			*p*
	Mean	SD	Range	Mean	SD	Range	
Forefoot Surface Area (%)	55.81	3.35	(48.00–64.10)	56.34	3.34	(50.10–66.30)	0.391
Midfoot Surface Area (%)	17.89	4.09	(4.20–23.70)	17.42	4.79	(1.60–23.60)	0.763
Rearfoot Surface Area (%)	26.30	2.45	(22.30–32.70)	26.26	2.80	(21.70–37.30)	0.697
Step Time (s)	201.38	611.94	(0.59–2360.00)	46.62	238.50	(0.55–1240.00)	0.309
Step Length (mm)	566.23	314.27	(300.34–1570.00)	463.99	93.24	(297.00–724.00)	0.566
Rearfoot Pressure (N/cm^2^)	23.17	8.60	(9.30–44.80)	23.80	8.16	(11.50–40.60)	0.607
Midfoot Pressure (N/cm^2^)	8.53	4.78	(2.50–21.80)	8.93	4.62	(1.90–19.70)	0.704
Forefoot Pressure (N/cm^2^)	18.06	7.00	(8.00–39.10)	18.60	6.77	(10.20–34.20)	0.666
Maximum Pressure Toe 1 (N/cm^2^)	33.15	24.65	(0.10–107.00)	34.73	23.09	(0.90–118.90)	0.636
Maximum Pressure Toes 2–5 (N/cm^2^)	8.74	7.10	(0.00–35.30)	9.26	6.97	(0.00–32.40)	0.680
**Maximum Pressure Metatarsal 1 (N/cm^2^)**	31.40	18.11	(8.50–84.20)	36.52	22.47	(7.40–110.90)	0.272
**Maximum Pressure Metatarsal 2 (N/cm^2^)**	56.91	32.79	(14.90–152.60)	61.28	31.71	(19.10–128.50)	0.469
**Maximum Pressure Metatarsal 3 (N/cm^2^)**	52.21	23.04	(12.80–125.00)	49.78	24.43	(16.50–130.70)	0.525
**Maximum Pressure Metatarsal 4 (N/cm^2^)**	38.68	18.34	(8.70–85.80)	38.04	19.71	(13.20–88.40)	0.781
**Maximum Pressure Metatarsal 5 (N/cm^2^)**	32.47	23.71	(6.30–115.50)	35.53	26.78	(4.20–129.00)	0.690
Maximum Pressure Midfoot (N/cm^2^)	12.40	8.87	(0.00–39.30)	14.88	9.64	(1.50–35.90)	0.248
Maximum Pressure Medial Heel (N/cm^2^)	44.68	19.61	(19.00–86.80)	45.89	18.79	(19.50–80.30)	0.711
Maximum Pressure Lateral Heel (N/cm^2^)	44.46	19.29	(14.00–105.50)	44.14	17.82	(16.10–84.10)	0.978
Rearfoot Impulse (N × s)	42,995.12	20,409.79	(18,062.40–99,469.22)	44,131.88	22,160.56	(0.00–94,199.04)	0.770
Midfoot Impulse (N × s)	109,238.68	77,059.70	(7683.84–359,595.36)	114,407.61	86,801.64	(3793.92–361,760.00)	0.982
Forefoot Impulse (N × s)	289,567.39	172,926.60	(71,672.16–1,131,974.40)	340,029.99	278,582.84	(92,659.20–1,604,050.56)	0.721
Rearfoot Impulse Percentage (%)	10.46	3.87	(3.90–18.40)	10.03	3.79	(0.00–20.60)	0.693
Midfoot Impulse Percentage (%)	24.45	12.26	(1.60–54.00)	23.79	12.99	(0.40–53.20)	0.813
Forefoot Impulse Percentage (%)	65.09	12.19	(31.00–84.10)	66.18	13.91	(37.60–99.60)	0.706

Comparisons of Forefoot Surface Area (%) and impulse percentages (%) were performed using the paired *t*-test (normal distribution). All other gait and pressure parameters were compared using the Wilcoxon signed-rank test (non-normal distribution).

## Data Availability

Data available on request due to privacy or ethical restrictions. The reason for this choice is that the dataset contains sensitive clinical information from participants. To protect their privacy and maintain confidentiality in accordance with data protection regulations, the raw data cannot be made publicly available in a repository. However, the data will be shared with the editorial office or researchers upon reasonable request for the purpose of internal evaluation or verification of the results.

## Abbreviations

The following abbreviations are used in this manuscript:

FPI	Foot Posture Index
MRC	Medical Research Council
MRI	Magnetic Resonance Imaging
SD	Standard Deviation
